# Combining virtual monoenergetic imaging and iterative metal artifact reduction in first-generation photon-counting computed tomography of patients with dental implants

**DOI:** 10.1007/s00330-023-09790-y

**Published:** 2023-06-07

**Authors:** Theresa Sophie Patzer, Andreas Steven Kunz, Henner Huflage, Philipp Gruschwitz, Pauline Pannenbecker, Saif Afat, Judith Herrmann, Bernhard Petritsch, Thorsten Alexander Bley, Jan-Peter Grunz

**Affiliations:** 1 Department of Diagnostic and Interventional Radiology, University Hospital Würzburg, Oberdürrbacherstraße 6, 97080 Würzburg, Germany; 2 Department of Diagnostic and Interventional Radiology, University Hospital Tübingen, Hoppe-Seyler-Str 3, 72076 Tübingen, Germany

**Keywords:** Artifact, Tomography, X-ray computed, Dental implants

## Abstract

**Objectives:**

While established for energy-integrating detector computed tomography (CT), the effect of virtual monoenergetic imaging (VMI) and iterative metal artifact reduction (iMAR) in photon-counting detector (PCD) CT lacks thorough investigation. This study evaluates VMI, iMAR, and combinations thereof in PCD-CT of patients with dental implants.

**Material and methods:**

In 50 patients (25 women; mean age 62.0 ± 9.9 years), polychromatic 120 kVp imaging (T3D), VMI, T3D_iMAR_, and VMI_iMAR_ were compared. VMIs were reconstructed at 40, 70, 110, 150, and 190 keV. Artifact reduction was assessed by attenuation and noise measurements in the most hyper- and hypodense artifacts, as well as in artifact-impaired soft tissue of the mouth floor. Three readers subjectively evaluated artifact extent and soft tissue interpretability. Furthermore, new artifacts through overcorrection were assessed.

**Results:**

iMAR reduced hyper-/hypodense artifacts (T3D 1305.0/−1418.4 versus T3D_iMAR_ 103.2/−46.9 HU), soft tissue impairment (106.7 versus 39.7 HU), and image noise (16.9 versus 5.2 HU) compared to non-iMAR datasets (*p *≤ 0.001). VMI_iMAR_ ≥ 110 keV subjectively enhanced artifact reduction over T3D_iMAR_ (*p *≤ 0.023). Without iMAR, VMI displayed no measurable artifact reduction (*p *≥ 0.186) and facilitated no significant denoising over T3D (*p *≥ 0.366). However, VMI ≥ 110 keV reduced soft tissue impairment (*p *≤ 0.009). VMI_iMAR_ ≥ 110 keV resulted in less overcorrection than T3D_iMAR_ (*p *≤ 0.001). Inter-reader reliability was moderate/good for hyperdense (0.707), hypodense (0.802), and soft tissue artifacts (0.804).

**Conclusion:**

While VMI alone holds minimal metal artifact reduction potential, iMAR post-processing enabled substantial reduction of hyperdense and hypodense artifacts. The combination of VMI ≥ 110 keV and iMAR resulted in the least extensive metal artifacts.

**Clinical relevance:**

Combining iMAR with VMI represents a potent tool for maxillofacial PCD-CT with dental implants achieving substantial artifact reduction and high image quality.

**Key Points:**

*• Post-processing of photon-counting CT scans with an iterative metal artifact reduction algorithm substantially reduces hyperdense and hypodense artifacts arising from dental implants.*

*• Virtual monoenergetic images presented only minimal metal artifact reduction potential.*

*• The combination of both provided a considerable benefit in subjective analysis compared to iterative metal artifact reduction alone.*

## Introduction

Maxillofacial evaluation of patients with dental implants by means of multidetector computed tomography (CT) poses a challenge in daily routine as artifacts impair radiological assessability of the dental implant itself as well as the circumjacent tissues. In consequence, detection of, e.g., tumors, inflammation, and osteolyses, may be hampered in the presence of metal implants [1].

Recent studies employing photon-counting detector (PCD) CT systems have reported high geometric dose-efficiency as well as the associated potential for substantial dose reduction. For one, PCDs are less susceptible to low-level image noise than energy-integrating detector (EID) builds [2–4]. In EID-CT, the total amount of energy deposited by the entirety of photons is integrated, including electronic noise. In contrast, PCD builds generate an electrical pulse proportional to each photon’s energy reaching the detector element. However, only pulses exceeding a predefined energy threshold are registered, effectively excluding low-level electronic noise [5, 6]. Integrating all PCD counts above the lowest energy threshold at 20 keV is defined as T3D by the vendor, comparing to conventional imaging in EID-CT [7]. Beam hardening is primarily caused by low-energy photons. Thus, apart from radiation dose reduction, PCD-CT scans may be less artifact-prone due to energy-weighting [8]. Furthermore, due to separate readout of smaller subpixels and the overcome necessity for optical separation, current PCD-CT systems allow for a superior spatial resolution with a minimal pixel size of 0.25 mm in ultrahigh-resolution mode [9, 10].

A plethora of different factors, including implant composition, influence the extent of metal artifacts [11–13]. Hypodense and hyperdense artifacts arise mainly due to scatter, undersampling, beam hardening, and photon starvation [14, 15]. While metal artifacts have been investigated predominantly for EID systems [16–18], concepts for metal artifact reduction comprised primarily the adjustment of acquisition and reconstruction parameters for a long time [11]. However, increased tube voltage and tube current bear the expense of increased radiation burden and are thus viewed critically in light radiation protection efforts. For one, spectral shaping via tin prefiltration has been shown to be a reliable strategy, additionally holding the potential for substantial dose reduction [19]. Despite being associated with the introduction of secondary artifacts and the possible alteration of image information [20, 21], post-processing techniques like iterative reconstruction methods are firmly anchored in clinical routine. Different vendors have offered iterative metal artifact reduction (iMAR) algorithms in EID-CT for years [22, 23]. Lately, iMAR has been adapted for PCD-CT, promising to improve its specific capability for metal artifact reduction. This iMAR algorithm is based on three different concepts for metal artifact reduction, namely normalized sinogram inpainting, beam hardening correction, and frequency-split metal artifact reduction. While normalized sinogram inpainting is designed to address artifacts in sinogram regions of high metal attenuation with the purpose to lower high-attenuation artifacts that occur tangential to high-contrast objects, beam hardening correction reduces artifacts in regions of minimal metal attenuation. Frequency-split technique helps to maintain image information that may be lost near the metal edge due to interpolation [20, 24]. Besides, multi-energy datasets allow for virtual monoenergetic image (VMI) reconstructions. Simulating images obtained from true monoenergetic acquisitions, high-kiloelectron volt VMIs are known to be less prone to beam hardening, creating potential for artifact reduction in the presence of different metal implants and devices [25, 26]. Moreover, recent studies investigating dual-layer and split-filter single-source dual-energy CT have reported superior metal artifact reduction for VMI combined with iMAR in patients with dental implants [27–29].

While the value of VMI and iterative reconstruction methods for metal artifact reduction has been demonstrated for dual-energy EID-CT, suchlike studies employing a PCD build are lacking. This investigation aims to evaluate a first-generation PCD system’s capability to reduce metal artifacts arising from dental implants using VMI and dedicated iterative MAR algorithms, as well as a combination of both.

## Methods

**Fig. 1:**
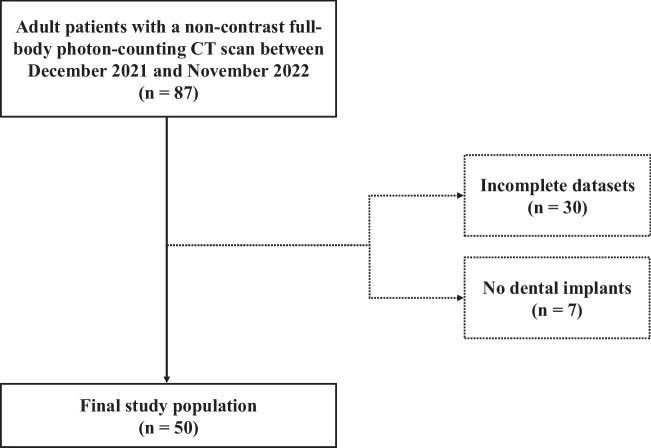
Flowchart illustrating study selection

This retrospective, single-center study was approved by the local institutional review board, which waived the requirement for informed consent. The investigation was conducted in the radiology department of a tertiary care university hospital. All patients receiving a clinically indicated non-contrast full-body PCD-CT scan for staging of multiple myeloma between December 2021 and November 2022 were retrospectively enrolled (*n *= 87). An age of or greater than 18 years and the presence of dental implants were mandatory for study inclusion. Lack of raw data for reconstruction of VMI and iMAR images in addition to conventional images represented exclusion criteria. A total of 50 patients were included in the final study group (Fig. 1).

### Imaging

All scans were performed employing a first-generation, cadmium-telluride-based PCD-CT system (Naeotom Alpha; Siemens Healthcare GmbH). Datasets were acquired as per clinical standard, with a detector collimation of 144 × 0.4 mm and a helical pitch factor of 1.2. Post-processing was carried out using dedicated software (syngo.via VB40B, Siemens Healthcare GmbH). Reformatting was conducted in axial orientation with a 512 × 512 pixel matrix and a field of view of 250 mm. A fourth-generation quantum iterative reconstruction algorithm (strength level 3; QIR, Siemens Healthcare GmbH) was used and a body imaging kernel (Qr40, Siemens Healthcare GmbH) was applied for scanner-side raw data reconstruction. Conventional polychromatic (T3D) and VMI images were acquired with a tube voltage of 120 kVp. Post-processing of spectral data allowed for VMI reconstructions, VMI images were computed at five different energy levels (40, 70, 110, 150, and 190 keV) covering the full kilovoltage range (40 to 190 keV). For VMI and T3D images, identical in-plane resolution was achieved with a predefined slice thickness of 2 mm and an increment of 1.5 mm. T3D and VMI images were reconstructed both with and without a dedicated iMAR algorithm (Siemens Healthcare GmbH). Window width and center were predefined at 400 and 40 HU, respectively, while readers were permitted to alter standard window settings. Image analysis was carried out using dedicated picture archiving and communication system software (Merlin, Phönix-PACS) and diagnostic monitors certified for clinical use (RadiForce RX660, EIZO).

### Objective image quality

**Fig. 2:**
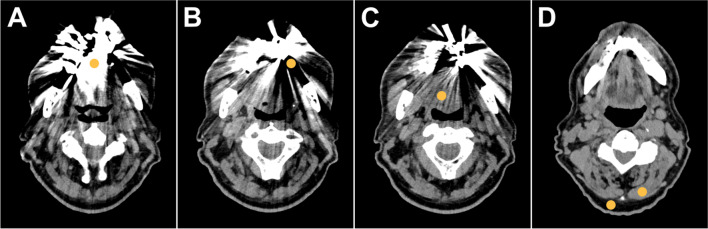
Region of interest placement in axial CT images for objective assessment of metal artifact intensity. Mean signal attenuation and standard deviation thereof were measured in the most hyperdense (**A**) and most hypodense artifacts (**B**) as well as in artifact-impaired soft tissue of the mouth floor (**C**). To correct for general lower image noise in high-keV reconstructions, additional measurements were performed in unimpaired muscle and subcutaneous fat tissue at the level of vertebra 2–3 (**D**)

For objective image analyses, a reader with 3 years of experience in musculoskeletal imaging placed regions of interest (ROIs) in the most pronounced hyperdense and hypodense artifacts, as well as in artifact-impaired soft tissue of the oral cavity. Thereafter, ROIs were positioned dorsally within the subcutaneous fat tissue at the level of the cervical vertebra 2 to 3 in a standardized manner. An additional ROI was placed in muscle tissue at the same level without artifact impairment for reference HU values. ROI placement was conducted in T3D images without iMAR and transferred to equivalent image positions in VMI and their counterparts with dedicated iMAR application (VMI_iMAR_). Exemplary ROI placement is shown in Fig. 2. ROI size was predefined to 10 mm^2^. Mean attenuation and standard deviation were recorded in Hounsfield units within each ROI. Due to generally higher image noise in low-kiloelectron volt reconstructions [30], corrected image noise was calculated as the difference of noise within artifact-impaired tissue and the reference lipid tissue. In order to account for differences in signal attenuation with varying kiloelectron volt values in similar fashion, corrected attenuation was calculated by subtracting the attenuation measured in artifact-free muscle tissue from the attenuation of artifact-impaired tissue.

### Subjective image quality

Subjective image assessment was independently performed by three radiologists (T.S.P., A.S.K., P.G.) with 3 to 9 years of skeletal imaging experience in blinded fashion and randomized order. The extent of hyperdense and hypodense artifacts was evaluated based on a 5-point scale (5 = absent/almost absent, 4 = minor, 3 = moderate, 2 = pronounced, 1 = severe). Diagnostic interpretability of soft tissue was also rated on the basis of a 5-point scale (5 = fully diagnostic, 4 = minor artifacts with marginal impairment of diagnostic interpretability, 3 = artifacts with impaired, mediocre diagnostic interpretability, 2 = artifacts with significantly impaired diagnostic interpretability, 1 = insufficient interpretability due to artifacts). Furthermore, overcorrection of existing artifacts and introduction of new artifacts were rated in binary manner compared to the respective image without additional iMAR.

### Statistical analysis

Dedicated software (SPSS Statistics 28, IBM) was used to carry out statistical analyses. Normal distribution of continuous variables was assessed with Kolmogorov-Smirnov and Shapiro-Wilk tests. If normally distributed, cardinal data are presented as mean ± standard deviation. For non-normally distributed and ordinal-scaled items, we report median values with interquartile ranges. Subjective and objective criteria of image quality were compared between reconstructions by means of Friedman’s two-way analysis of variance by ranks with pairwise post hoc tests. *p* values were corrected for multiple comparisons using the Bonferroni procedure. Dichotomous items, i.e., the introduction of new or aggravation of pre-existing artifacts through application of iMAR, were compared using Cochran’s *Q* test as a repeated measures ANOVA with Bonferroni-corrected pairwise post hoc testing. To assess inter-reader reliability, the intraclass correlation coefficient (ICC) was computed based on absolute agreement of single measures in a two-way random effects model. ICC results were interpreted following Koo and Li [31]; ICC > 0.90 = excellent; 0.75 – 0.90 = good; 0.50 – 0.75 = moderate; < 0.50 = poor reliability. Reader agreement for dichotomous variables (i.e., the overcorrection by iMAR application) was analyzed by calculating Krippendorff’s alpha (⍺). *p* values of ≤  0.05 were considered to indicate statistical significance.

## Results

A total of 50 patients (male/female: 25/25) with an average age of 62.0 ± 9.9 (range 45 – 85) were included in the analysis.

### Objective image quality

Irrespective of kiloelectron volt level, reconstructions with iMAR correction provided less pronounced hyperdense artifacts than their counterparts without dedicated iMAR application (all *p *< 0.001). Compared with polychromatic T3D imaging, only VMI at 190 keV resulted in a significant reduction of hyperdense artifacts (with iMAR: *p *= 0.011; without iMAR: *p *= 0.035).

The extent of hypodense artifacts was lower in iMAR datasets compared to the respective non-iMAR reconstructions, notwithstanding the kiloelectron volt level (all *p *< 0.001). Employing iMAR, only VMI at 190 keV allowed for hypodense artifact reduction compared to T3D (*p *= 0.002), while the artifact intensity was comparable for VMI_iMAR_ at 150 keV or less (all *p *≥ 0.081). No significant difference was ascertained between the intensity of hypodense artifacts in standard T3D and VMI (*p *> 0.999).

For ≥ 70 keV, VMI_iMAR_ provided less artifact impairment in adjacent soft tissue than standard VMI (all *p *≤ 0.001). Only VMI_iMAR_ at 40 keV displayed stronger artifacts in soft tissue than T3D_iMAR_ (*p *= 0.002). Artifact intensity between VMI_iMAR_ at 70 keV and T3D_iMAR_ was similar (*p *> 0.999). Soft tissue impairment was considerably lower in VMI ≥ 110 keV compared to T3D (all *p *≤ 0.009). Accordingly, VMI_iMAR_ ≥ 110 keV allowed for substantially less artifact impairment in soft tissue than T3D_iMAR_ (all *p *≤ 0.002).

**Fig. 3:**
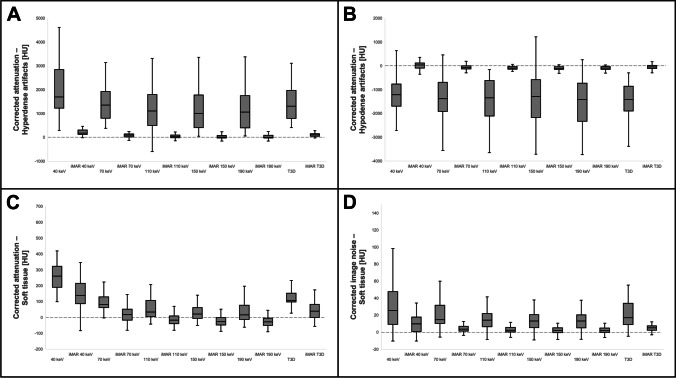
Boxplots illustrating corrected attenuation for hyperdense (**A**) and hypodense artifacts (**B**), artifact-impaired soft tissue (**C**) as well as corrected image noise (**D**). Note: solid line within the box = median; edges of the boxes = upper/lower quartiles; extremes of whiskers = minimum and maximum values within 1.5-fold of interquartile range. iMAR, iterative metal artifact reduction; VMI, virtual monoenergetic image reconstructions; T3D, conventional images

Regardless of kiloelectron volt level, reconstructions with iMAR correction exhibited less image noise than their non-iMAR counterparts (all *p *≤ 0.001). Compared to polychromatic T3D images, no significant noise reduction could be achieved through VMI between 40 and 190 keV with or without iMAR post-processing (all *p *≥ 0.366). Boxplot diagrams summarizing objective image quality assessment are provided in Fig. 3. Table 1 displays the detailed results of objective image quality assessment.

**Table 1:** Objective assessment of artifact reduction and surrounding tissue

	Corrected attenuation (HU)			Corrected noise (HU)
	Hyperdense artifacts	Hypodense artifacts	Artifact-impaired soft tissue	Artifact-impaired soft tissue
	Median (interquartile range)			
T3D	1305.0 (1134.7)	(−) 1418.4 (998.7)	106.7 (49.7)	16.9 (23.7)
40 keV	1698.3 (1584.9)	(−) 1213.4 (898.6)	259.9 (129.9)	25.5 (36.3)
70 keV	2352.1 (1105.0)	(−) 1384.2 (1167.6)	81.2 (62.0)	14.7 (20.8)
110 keV	1104.1 (1260.0)	(−) 1349.0 (1447.3)	33.3 (88.5)	14.3 (14.9)
150 keV	1004.1 (1359.7)	(−) 1284.9 (1518.6)	21.8 (54.3)	13.2 (14.9)
190 keV	1061.4 (1337.0)	(−) 1413.5 (1517.2)	16.2 (77.9)	13.1 (14.7)
T3D_iMAR_	103.2 (95.0)	(−) 46.9 (127.9)	39.7 (80.0)	5.2 (4.6)
40 keV_iMAR_	175.4 (166.9)	39.1 (200.2)	138.2 (123.0)	9.9 (16.1)
70 keV_iMAR_	91.6 (107.8)	(−) 68.8 (123.1)	17.8 (64.3)	3.6 (5.1)
110 keV_iMAR_	30.2 (93.4)	(−) 92.5 (104.1)	(−) 17.7 (47.0)	2.4 (4.8)
150 keV_iMAR_	4.3 (95.8)	(−) 96.9 (108.9)	(−) 26.5 (45.0)	2.1 (5.5)
190 keV_iMAR_	2.8 (95.8)	(−) 95.3 (110.1)	(−) 28.2 (43.9)	1.9 (5.0)
T3D				
vs. 40 keV	> 0.999	> 0.999	> 0.999	> 0.999
vs. 70 keV	> 0.999	> 0.999	> 0.999	> 0.999
vs. 110 keV	> 0.999	> 0.999	**0.009**	> 0.999
vs. 150 keV	0.283	> 0.999	**< 0.001**	> 0.999
vs. 190 keV	**0.035**	> 0.999	**< 0.001**	> 0.999
Non-iMAR vs. iMAR				
T3D vs. T3D_iMAR_	**< 0.001**	**< 0.001**	**0.002**	**< 0.001**
40 keV vs. 40 keV_iMAR_	**< 0.001**	**< 0.001**	> 0.999	**0.001**
70 keV vs. 70 keV_iMAR_	**< 0.001**	**< 0.001**	**< 0.001**	**< 0.001**
110 keV vs. 110 keV_iMAR_	**< 0.001**	**< 0.001**	**< 0.001**	**< 0.001**
150 keV vs. 150 keV_iMAR_	**< 0.001**	**< 0.001**	**0.001**	**< 0.001**
190 keV vs. 190 keV_iMAR_	**< 0.001**	**< 0.001**	**0.001**	**< 0.001**
T3D_iMAR_				
vs. 40 keV_iMAR_	> 0.999	> 0.999	**0.002**	> 0.999
vs. 70 keV_iMAR_	> 0.999	> 0.999	> 0.999	> 0.999
vs. 110 keV_iMAR_	> 0.999	> 0.999	**0.002**	> 0.999
vs. 150 keV_iMAR_	0.198	0.081	**< 0.001**	> 0.999
vs. 190 keV_iMAR_	**0.011**	**0.002**	**< 0.001**	0.366

*p* values indicating statistical significance highlighted in bold. *iMAR* iterative metal artifact reduction

### Subjective image quality

**Fig. 4:**
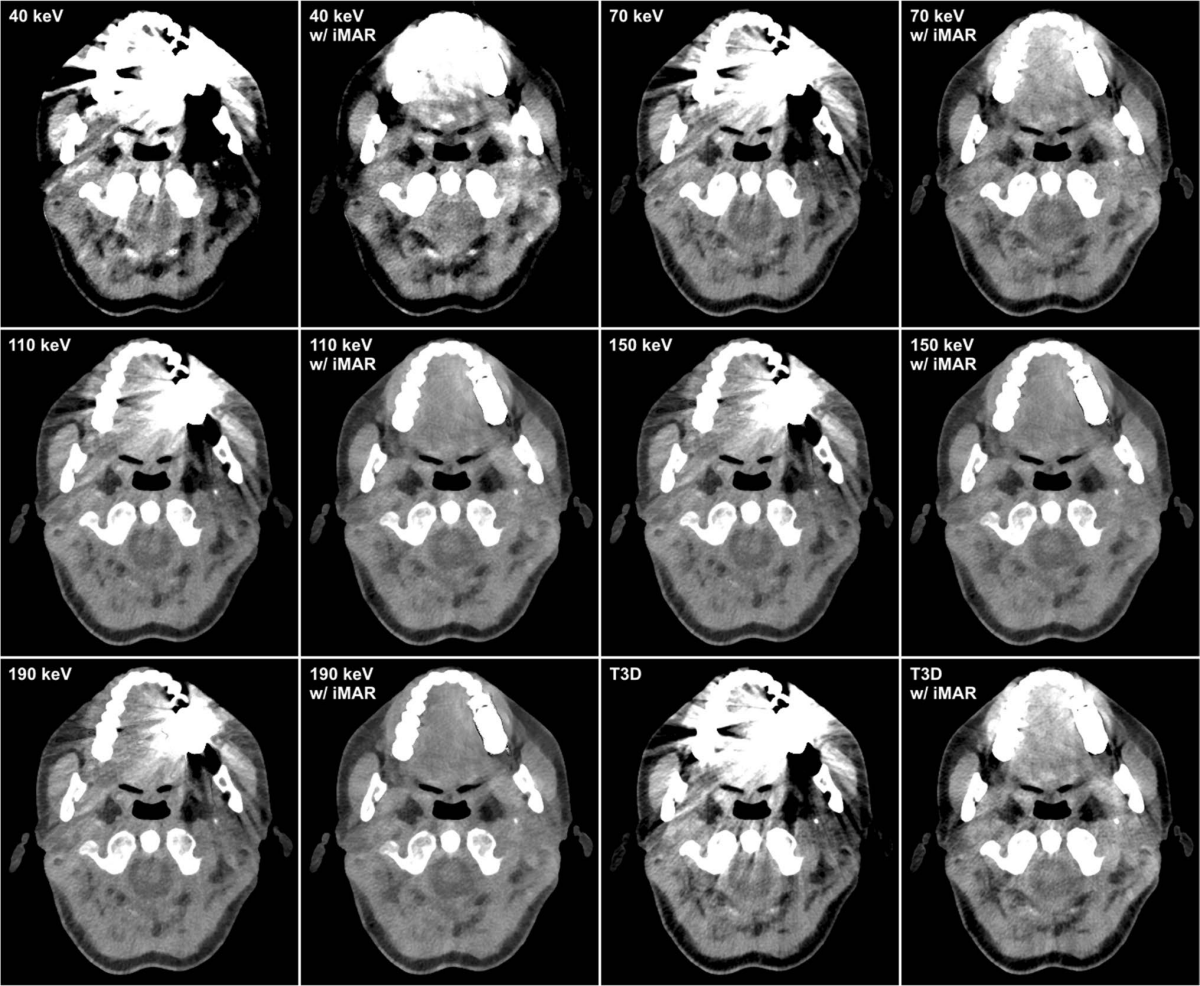
Extent of hyperdense and hypodense artifacts as well as severity of soft tissue impairment on representative axial slices in the same patient with a dental implant in the left maxilla for conventional (T3D) and virtual monoenergetic images (VMI) with and without addition of iterative metal artifact reduction

The extent of hyperdense and hypodense artifacts, as well as the severity of soft tissue impairment, is shown on representative axial slices for T3D and VMI images with and without employment of the iMAR algorithm (Fig. 4). Cumulative results for subjective assessment of artifact reduction and interpretability of surrounding tissue are summarized in Table 2.

**Table 2:** Pooled subjective assessment of artifact reduction and surrounding tissue

	Hyperdense artifacts	Hypodense artifacts	Soft tissue assessability
	Median ratings of three radiologists (interquartile range)		
T3D	1 (1–1)	1 (1–1)	1 (1–1)
40 keV	1 (1–1)	1 (1–1)	1 (1–1)
70 keV	1 (1–1)	1 (1–1)	1 (1–1)
110 keV	1 (1–1)	1 (1–1)	1 (1–1)
150 keV	1 (1–1)	1 (1–2)	1 (1–1)
190 keV	1 (1–2)	1 (1–2)	1 (1–1)
T3D_iMAR_	2 (1–2)	3 (2–3)	2 (1–2)
40 keV_iMAR_	1 (1–2)	2 (2–3)	1 (1–1)
70 keV_iMAR_	2 (1–3)	3 (2–3)	2 (1–2)
110 keV_iMAR_	2 (2–3)	3 (3–4)	2 (2–3)
150 keV_iMAR_	3 (2–3)	3 (3–4)	3 (2–4)
190 keV_iMAR_	3 (3–4)	4 (3–4)	3 (3–4)
T3D			
vs. 40 keV	> 0.999	> 0.999	> 0.999
vs. 70 keV	> 0.999	> 0.999	> 0.999
vs. 110 keV	> 0.999	> 0.999	> 0.999
vs. 150 keV	0.862	> 0.999	> 0.999
vs. 190 keV	0.186	> 0.999	> 0.999
Non-iMAR vs. iMAR			
T3D vs. T3D_iMAR_	**< 0.001**	**< 0.001**	**< 0.001**
40 keV vs. 40 keV_iMAR_	**0.001**	**< 0.001**	0.964
70 keV vs. 70 keV_iMAR_	**< 0.001**	**< 0.001**	**< 0.001**
110 keV vs. 110 keV_iMAR_	**< 0.001**	**< 0.001**	**< 0.001**
150 keV vs. 150 keV_iMAR_	**< 0.001**	**< 0.001**	**< 0.001**
190 keV vs. 190 keV_iMAR_	**< 0.001**	**< 0.001**	**< 0.001**
T3D _iMAR_			
vs. 40 keV_iMAR_	**< 0.001**	**0.023**	**< 0.001**
vs. 70 keV_iMAR_	> 0.999	> 0.999	> 0.999
vs. 110 keV_iMAR_	**0.005**	**0.023**	**< 0.001**
vs. 150 keV_iMAR_	**< 0.001**	**< 0.001**	**< 0.001**
vs. 190 keV_iMAR_	**< 0.001**	**< 0.001**	**< 0.001**
ICC (95% CI)	0.707 (0.582–0.788)	0.802 (0.777–0.825)	0.804 (0.774–0.831)

*p* values indicating statistical significance highlighted in bold. *iMAR* iterative metal artifact reduction, *ICC* intraclass correlation coefficient (two-way random effects model based on absolute agreement), *CI* confidence interval

#### Hyperdense artifacts

Pooled ratings by three radiologists indicated substantial reduction of hyperdense artifacts in reconstructions with iMAR application compared to the respective non-iMAR datasets (all *p *≤ 0.001). While VMI_iMAR_ 40 keV was considered to feature stronger artifacts in adjacent soft tissue than T3D_iMAR_ (*p *< 0.001), artifact intensity of 70 keV was deemed similar to T3D_iMAR_ (*p *> 0.999). All analyzed VMI_iMAR_ ≥ 110 keV allowed for artifact reduction superior to T3D_iMAR_ (all *p *≤ 0.005). Without iMAR application, no substantial difference was established between VMI and polychromatic T3D regarding hyperdense artifacts (all *p *≥ 0.186).

#### Hypodense artifacts

According to subjective image analysis, a substantial reduction of hypodense artifacts could be achieved in iMAR reconstructions compared to their counterparts without additional iMAR application (all *p *< 0.001). While VMI_iMAR_ at 40 keV showed a higher extent of hypodense artifacts than T3D_iMAR_ (*p *= 0.023), no differences of artifact intensity were ascertained between VMI_iMAR_ at 70 keV and T3D_iMAR_ (*p *> 0.999). In contrast, substantial artifact reduction could be realized in all VMI_iMAR_ ≥ 110 keV compared to T3D_iMAR_ (all *p *≤ 0.023). Ratings of hypodense artifact intensity were comparable between polychromatic T3D and standard VMI (all *p *> 0.999).

#### Soft tissue impairment

For T3D and all VMI ≥ 40 keV, pooled ratings indicated a substantial improvement of soft tissue interpretability in reconstructions with iMAR application compared to the respective non-iMAR images (all *p *< 0.001). Compared to T3D_iMAR_, ratings for artifacts in adjacent soft tissue were higher for 40 keV VMI_iMAR_ (*p *< 0.001), while soft tissue impairment in VMI_iMAR_ at 70 keV was perceived to be similar to T3D_iMAR_ (*p *> 0.999). Soft tissue assessability in all analyzed VMI_iMAR_ ≥ 110 keV was better than that in T3D_iMAR_ (all *p *≤ 0.001). No substantial difference was ascertained between VMI and standard polychromatic T3D in non-iMAR reconstructions (all *p *≥ 0.999).

#### New artifacts/overcorrection

**Fig. 5:**
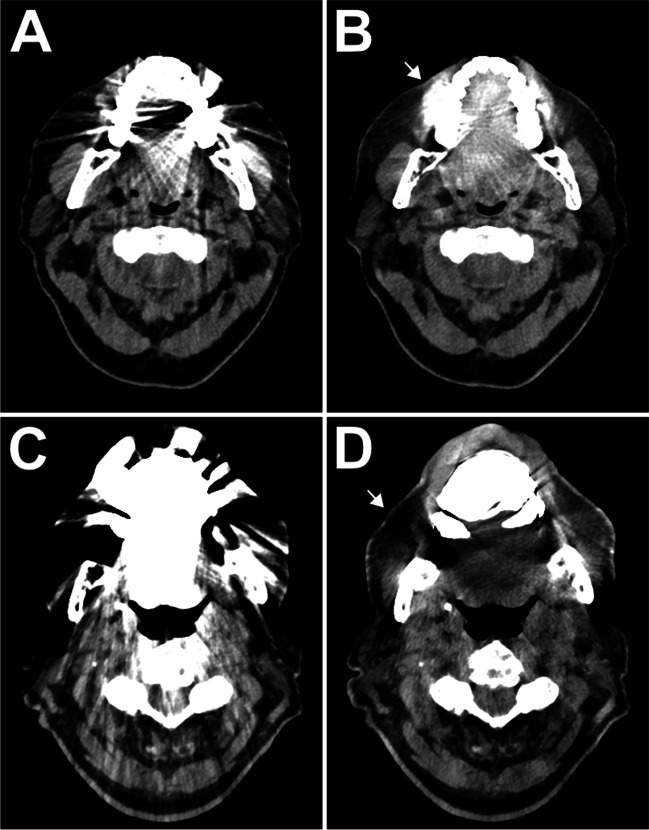
Introduction of new hyperdense artifacts in a patient with maxillary implants (**A** conventional T3D; **B** T3D_iMAR_). In another patient, overcorrection by iterative metal artifact reduction led to a loss of signal in periosseous soft tissue (**C** conventional T3D; **D** T3D_iMAR_). Arrows indicate the newly introduced artifacts

iMAR introduced new or aggravated preexisting artifacts at 40 keV in stronger fashion than in polychromatic T3D_iMAR_ (*p *= 0.009), whereas no substantial difference was determined for VMI_iMAR_ at 70 keV and T3D_iMAR_ (*p *> 0.999). All VMI_iMAR_ ≥ 110 keV resulted in less overcorrection than T3D_iMAR_ (all *p *≤ 0.001). Figure 5 displays two examples of newly introduced artifacts in representative axial slices. Absolute and relative frequencies of new artifacts and iMAR overcorrection are provided in Table 3. Inter-reader reliability for assessment of hyperdense artifacts was moderate, indicated by an ICC of 0.707 (95% confidence interval of 0.582–0.788). Reliability for both evaluation of hypodense artifacts (ICC = 0.802 [0.777–0.825]) and soft tissue impairment by artifacts (ICC = 0.804 [0.774–0.831]) was good. Agreement between readers for iMAR overcorrection was high (⍺ = 0.938 [0.910–0.963]).

**Table 3:** Subjective assessment of new artifacts and iMAR overcorrection. Results are provided as absolute and relative frequencies

	*n* (percentage)	*p* value
T3D_iMAR_	44 (29.3%)	
vs. 40 keV_iMAR_	62 (41.3%)	**0.009**
vs. 70 keV_iMAR_	45 (30.0%)	> 0.999
vs. 110 keV_iMAR_	20 (13.3%)	**< 0.001**
vs. 150 keV_iMAR_	20 (13.3%)	**< 0.001**
vs. 190 keV_iMAR_	18 (12.0%)	**< 0.001**

*p* values indicating statistical significance highlighted in bold. *iMAR* iterative metal artifact reduction

## Discussion

This study investigated the metal artifact reduction potential of photon-counting detector CT in patients with dental implants using virtual monoenergetic imaging and dedicated iterative reconstruction algorithms, as well as a combination of both. Evaluating 50 examinations on a first-generation photon-counting CT scanner, subjective and objective image analysis indicated the remarkable artifact reduction potential of iMAR-supported reconstructions compared to the respective non-iMAR datasets. The combination of VMI ≥ 110 keV and iMAR provided a considerable benefit in subjective analysis compared to polychromatic T3D imaging with iMAR, whereas VMI without iMAR displayed only minimal artifact-reducing effects.

In synopsis with the current literature on EID-CT systems, we confirm the postulated reduction of hyperdense and hypodense artifacts, as well as decreased image noise in iMAR reconstructions compared to the respective non-iMAR images, irrespective of kiloelectron volt level. Furthermore, our results revealed that soft tissue impairment was substantially lowered for all VMI at greater than 40 keV with dedicated iMAR application compared to their equivalents without. While Schmidt et al [27] reported slightly improved artifact reduction when combining VMI with iMAR at 100 keV in a split-filter dual-energy EID-CT, the present analysis on PCD-CT data implies a similar advantage over conventional imaging only for VMI_iMAR_ at 190 keV. Otherwise, the combination of VMI and iMAR showed no benefit in metal artifact reduction compared with T3D_iMAR_ in our objective image analysis. However, VMI_iMAR_ at 110 keV or greater yielded favorable results in subjective image analysis, though.

In PCD-CT, high-kiloelectron volt thresholds are known to be less susceptible to beam hardening effects [5, 32, 33]. On the downside, low-energy photons do not contribute to image information when employing high-energy thresholds, which are thus associated with increased image noise and reduced radiation dose efficiency [8, 34]. While recent studies confirmed the effectiveness of high-kiloelectron volt VMI for reduction of beam hardening in EID-CT [26, 35] and PCD-CT [36], we could only demonstrate this effect for VMI of 190 keV, while VMI alone had no relevant artifact-reducing effect. In contrast, Anhaus et al [37] suggested that high-kiloelectron volt reconstructions bear no advantage for metal implants with high atomic numbers such as dental hardware. This is in line with Schmidt et al [27] and Laukamp et al [28], who investigated metal artifact reduction techniques in EID-CT and postulated that VMI alone had no substantial impact on hyperdense and hypodense artifacts in comparison to standard images. On the other hand, iMAR algorithms are well-established in clinical EID-CT routine and have shown to be a powerful tool for metal artifact reduction in various imaging tasks employing different scanner types [37–39]. In general, iMAR algorithms are specific to a particular scanner type, impeding direct comparisons between vendors and technical concepts such as EID-CT and PCD-CT [26, 28]. Nonetheless, our results suggest a certain degree of transferability between the detector technologies.

Even though iterative reconstruction algorithms represent a potent approach for metal artifact reduction, these iMAR-enhanced images ought not to fully replace conventional images but much rather should be considered an add-on due to potentially newly introduced or aggravated image artifacts. Regarding suchlike changes to images due to iMAR reformatting, our results concur with the current EID-CT literature [23, 40]. While VMI_iMAR_ at 40 keV introduced new or featured stronger artifacts in some cases, VMI_iMAR_ at 110 keV or greater resulted in less suchlike alterations. Addressing these drawbacks, Leng et al [34] and Zhou et al [8] suggested a combination of high-kiloelectron volt imaging and additional tin-prefiltration for improved metal artifact reduction in PCD-CT. However, as spectral shaping approaches harden the X-ray beam and increase the percentage of high-energy photons, soft tissue contrast, among others, is known to be impaired [41]. Future studies analyzing the value of tin-prefiltration in PCD-CT for metal artifact reduction are mandated.

Some limitations ought to be mentioned regarding this retrospective study. First, 50 CT examinations constitute a relatively small sample size. Only patients receiving a scan without contrast enhancement were included as high-kiloelectron volt reconstructions are considered unfavorable in combination with contrast agents due to loss of image contrast. Second, since visualization of artifact-adjacent soft tissue was our primary focus, the evaluation of teeth and bone was not in the scope of this study. Third, the effect of different implant placements and post-processing filtering was not evaluated [42]. Furthermore, dental implant composition was unknown, which may have affected the comparability of resulting artifacts and the efficacy of metal artifact reduction. Fourth, no iMAR solutions for PCD-CT are currently available from other vendors; hence, no inter-vendor comparisons could be performed. Fifth, we evaluated the artifact extent by measuring the attenuation in the most pronounced hypodense and hyperdense artifacts in addition to calculating the corrected image noise. Other studies quantified image noise employing dedicated algorithms [43–45].

To conclude, while VMI alone presented only minimal metal artifact reduction potential, post-processing using iMAR enabled a substantial reduction of hyperdense and hypodense artifacts. The combination of VMI ≥ 110 keV and iMAR resulted in the least extensive metal artifacts in patients with dental implants.

## Abbreviations

CT: Computed tomography
EID: Energy-integrating detector
HU: Hounsfield units
iMAR: Iterative metal artifact reduction
PCD: Photon-counting detector
ROI: Region of interest
VMI: Virtual monoenergetic imaging

## Declarations

### Guarantor

The scientific guarantor of this publication is Theresa Patzer (Patzer_T@ukw.de).

### Statistics and biometry

One of the authors (Jan-Peter Grunz) has significant statistical expertise and no complex statistical methods were necessary for this paper.

### Methodology

- retrospective
- observational
- performed at one institution
